# Electro-Optical Properties of Low-Temperature Growth Indium-tin-oxide Nanowires Using Polystyrene Spheres as Catalyst

**DOI:** 10.1186/s11671-016-1342-8

**Published:** 2016-03-09

**Authors:** Qiang Li, Zhina Gong, Yufeng Li, Hao Liu, Lungang Feng, Shuo Liu, Feng Yun

**Affiliations:** Key Laboratory of Physical Electronics and Devices of Ministry of Education and Shaanxi Provincial Key Laboratory of Photonics & Information Technology, Xi’an Jiaotong University, Xi’an, Shaanxi People’s Republic of China; Solid-State Lighting Engineering Research Center, Xi’an Jiaotong University, Xi’an, Shaanxi People’s Republic of China; Shaanxi Supernova Lighting Technology Co., Ltd, Xi’an, Shaanxi China

**Keywords:** Indium-tin-oxide nanowires, Electron-beam evaporation, Polystyrene spheres

## Abstract

Polystyrene sphere was chosen as a catalyst to fabricate indium-tin-oxide (ITO) nanowires (NWs) with a low-temperature (280–300 °C) electron-beam deposition process, bearing high purity. The ITO NWs with diameter of 20–50 nm and length of ~2 um were obtained. X-ray diffraction and high-resolution transmission electron microscope show high crystal quality. The transmittance is above 90 % at a wavelength 400 nm or more, superior to the ITO bulk film. Owing to the unique morphology gradient of the ITO NWs, the effective refractive index of ITO NWs film is naturally graded from the bottom to the top. The ITO NWs have been used on LED devices (*λ* = 450 nm), which improved the light output power by 31 % at the current of 150 mA comparing to the one without NWs and did not deteriorate the electrical properties. Such ITO NWs open opportunity in LED devices to further improve light extraction efficiency.

## Background

Indium-tin-oxide (ITO) film with high electrical conductivity and high transmittance in the visible light region is generally used in optoelectronic devices. Recently, the nanostructures of semiconducting metal oxides, such as nanorods, nanowires, and nanobelts, have attracted much attention because of their high surface-volume ratio and excellent optical/electrical properties [1–5]. Although there have been reports on the synthesis of nanostructures of ITO, most of them were grown with high-cost, low-purity, or complex processes [6–12].

In order to obtain the nanostructures with controlled size, high uniformity, and stable process, catalytic method is typically used, in which the size of the nanostructure is controlled by noble metal particles as catalyst. For example, ITO nanowires (NWs) have been grown using Au as catalyst, based on vapor-liquid-solid (VLS) method [3, 13], electron-beam deposition [14, 15], or radio frequency (RF) magnetron sputtering deposition [16,17]. However, there are some drawbacks to these methods. The VLS process needs high temperature (800~900 °C). The oblique-angle electron-beam evaporation is difficult to control cause of the shadow effect. The sputtering method is limited by the RF power and the complicated process. In addition, a common issue of these methods is the complete removal of catalyst (e.g., Au particles), impeding the fabrication of high-purity ITO NWs. Therefore, new catalysts and new processes are in need to address this issue. The polymer monodisperse microspheres with large specific surface area, strong adsorption, aggregation, and surface reaction ability have been used in the field of medical immunology, biological engineering, chemical industry, and microelectronics [18–21]. In this study, we report a method to fabricate high-quality ITO NWs, by means of electron-beam deposition with organic macromolecular material as catalyst. This method is a novel method for the preparation of ITO NWs using polystyrene (PS) as catalyst at a low temperature (280–300 °C), which has the advantages of low-cost, facile and efficient operation. This method prepares PS spheres on substrate by self-assembly, followed by the use of electron-beam evaporation to fabricate ITO NWs via PS, and then removes the PS by annealing, thereby obtained ITO NWs with better crystal quality.

## Methods

**Fig. 1:**
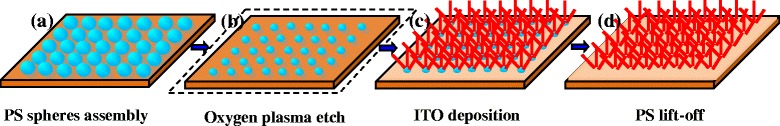
Fabrication procedure of ITO nanowires. **a** PS assembly on p-GaN or quartz wafer. **b** PS size reduced by oxygen plasma etching. **c** ITO NWs deposited on samples. **d** PS lift-off and ITO NWs film fabricated

Figure 1 is the schematic diagram illustrating the growth of ITO NWs. Firstly, a hexagonally ordered monolayer template of PS (size of 670 nm) spheres was coated on quartz or GaN wafer (2 in.) by self-assembly [22] (Fig. 1a). The PS spheres were then etched by oxygen plasma for 200 s to modify the morphology and to reduce the diameter of PS spheres to ~500 nm while increasing the distance between PS spheres (Fig. 1b). After that, ITO source (In:Sn = 9:1) was deposited on the template by electron-beam evaporation at a deposition rate of 0.1 nm/s for 15–20 min, with the chamber temperature stabilizing at 280–300 °C and pressure less than 5 × 10^−4^ Pa (Fig. 1c). At last, PS material was removed by annealing, using the property of PS decomposition at high temperature. The sample was annealed at 470 °C for 10–15 min under the nitrogen condition. So, the ITO NWs have been fabricated purely, shown as Fig. 1d.

In order to get more uniform distribution of PS spheres, the ICP etching is used to adjust the morphology (the scanning electron microscopy images are Appendix 1). The PS spheres without ICP etching can also serve as the catalyst to grow ITO NWs. The etching step is not necessarily to be formed. The chamber temperature was kept at 280–300 °C to ensure the surface of PS spheres remain in a molten state. For comparison, the conventional ITO films were deposited on the p-GaN surface and a quartz wafer without PS spheres in the same condition.

## Results and Discussion

**Fig. 2:**
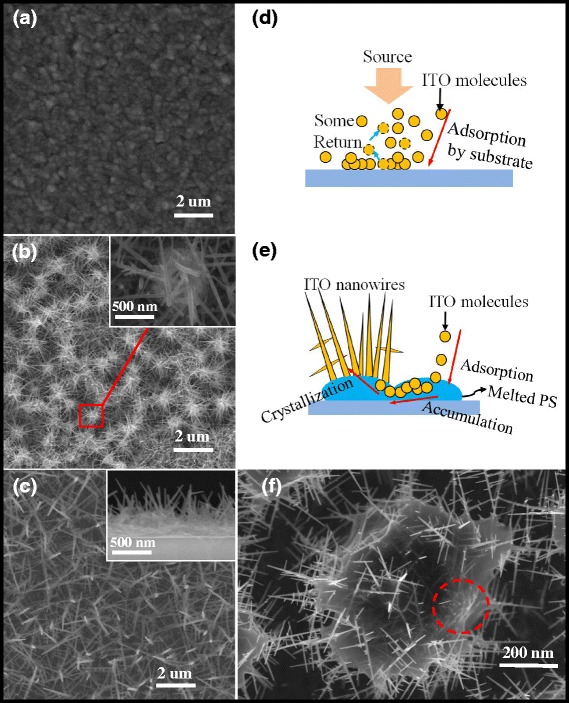
**a** Top view SEM image of conventional ITO film. **b** Top view SEM images of the ITO NWs film with PS. The *inset* shows the magnified view of circled ITO NWs on a separate PS sphere. **c** Top view SEM images of the ITO NWs film without PS, using high temperature to remove the PS sphere. The *inset* shows the cross-sectional SEM image of the ITO NWs fabricated on GaN substrate. **d** The schematic diagram of ITO deposition without PS spheres, **e** with PS spheres at 300 °C. **f** The SEM image of ITO NWs growth process in a melted PS sphere

Figure 2 shows the top view scanning electron microscopy (SEM) images of the ITO NWs fabricated on p-GaN substrate. The conventional ITO film (thickness of 80 nm) deposited on the surface without PS spheres is shown in Fig. 2a. We can see there are no NWs on the substrate. The one (Fig. 2b) with PS sphere is deposited with ITO under the same condition; the ITO NWs have been fabricated. It indicates that the catalyst of polystyrene is crucial for the formation of ITO NWs. In the process of ITO deposition, the chamber temperature is 300 °C, when the PS spheres begin to melt. Due to the larger surface energy of melting PS, the mixed molecules of In_2_O_3_ and SnO_2_ were adsorbed into molten PS spheres continuously. According to the vapor-liquid-solid (VLS) mechanism, the crystallization began to form nanowires, when the molecules were supersaturated in PS spheres. ITO NWs have been grown on PS spheres, showing “sea urchin” morphology as the insert figure in Fig. 2b. The morphology of ITO NWs post the removal of the PS by annealing is showed as Fig. 2c. We can see that the formed ITO NWs are compactly knitted and the morphology of ITO NWs has not changed after removing the PS spheres. The insert image in Fig. 2c shows cross-sectional SEM image of the ITO NWs fabricated on GaN, showing that the NWs have a tendency to grow perpendicularly above the surface of substrate. The diameter of the prepared ITO NWs is 20–50 nm, and the length can reach ~2 um.

Without PS spheres as the catalyst, some ITO molecules are adsorbed and get together on the substrate, while another portion of the molecules is bounced back, shown in Fig. 2d. The surface adsorption capacity of substrate is relatively small, and the substrate surface is lack of induced materials. Under such conditions, the ITO material cannot grow along the fixed crystal, so the NWs cannot be fabricated. When the substrate is coated with PS spheres, the PS is in the molten state under the temperature of 300 °C, and the surface adsorption energy is larger. In this case, the ITO molecules and In-Sn alloy are adsorbed preferentially by the melted PS spheres. In-Sn alloy keeps its liquid state in PS, and then, the impinging atoms have been absorded by which, because of their higher sticking coefficient. Nucleation occurs at the droplet-PS interface, resulting in the ITO NWs growth, shown in Fig. 2e. Figure 2f is the SEM image of ITO NWs growth process in a melted PS sphere. It is seen that NWs are grown on PS sphere, and long NWs begin to intertwine with the neighboring ones. Because of the high transparency of melted PS, we can clearly see that ITO molecules are adsorbed and agglomerated in PS from the red circle area (the surface is slightly raised, and the interior is filled with granular material.), and the morphology of crystallization at the beginning stage can also be observed.

**Fig. 3:**
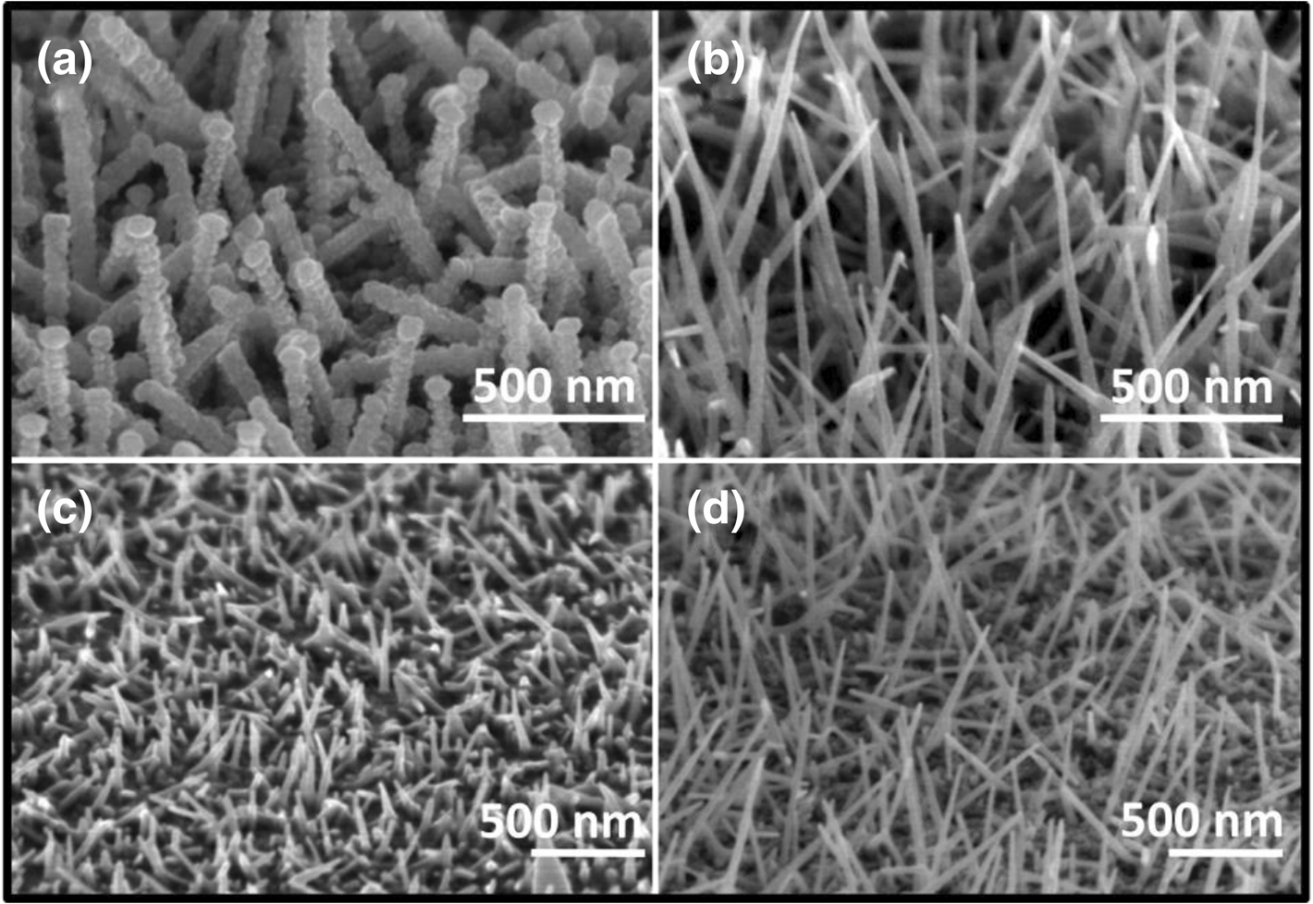
The SEM images of ITO NWs prepared by **a** 200 nm and **b** 670 nm PS spheres for 20 min. The morphology of ITO NWs prepared for **c** 5 min and **d** 15 min with same size diameter PS spheres

The diameter and length of ITO NWs can be controlled by using different diameters of PS spheres and the deposition time. On the one hand, two GaN substrates, with 200- and 670-nm diameter of PS spheres, were deposited ITO under the same condition (deposition rate of 0.1 nm/s, chamber temperature stabilizing at 300 °C and pressure less than 5 × 10^−4^Pa) for 20 min. The ITO NWs had large diameter (~50 nm) and rough surface, when they were prepared by 200-nm spheres (Fig. 3a). And, all the prepared NWs had small diameter (~20 nm) and needle shape by using 670-nm spheres (Fig. 3b). On the other, two samples with same size of PS spheres (670 nm) were deposited for 5 and 15 min, respectively. The results are shown in Fig. 3c, d. The average growth length of ITO NWs is ~200 nm in 5 min and can reach ~1 μm in 15 min.

**Fig. 4:**
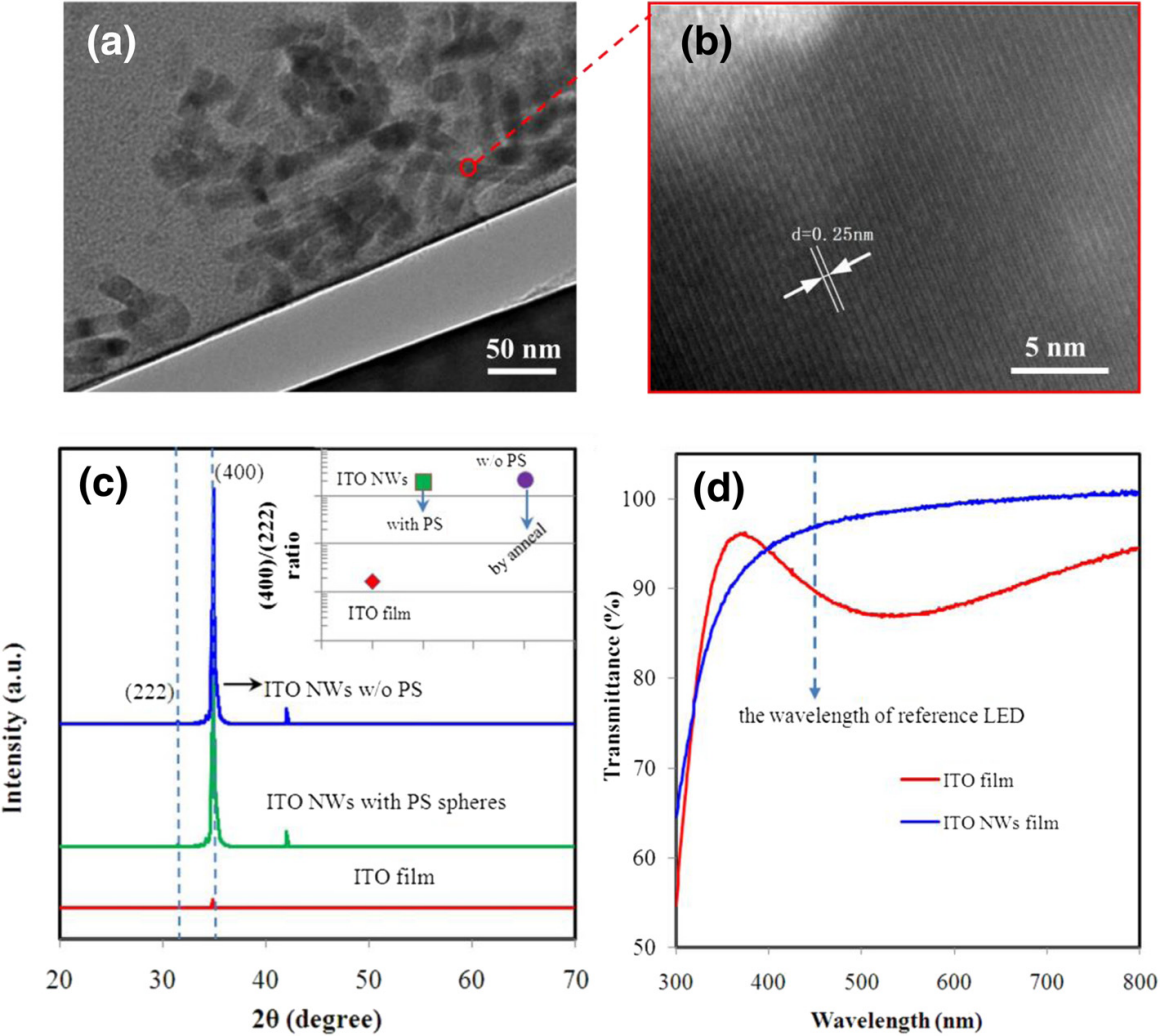
**a** The TEM image and **b** the HRTEM image of the NWs. **c** The XRD spectra of ITO film and ITO NWs film. The *inset* is the intensity ratio of (400)/(222) peaks. **d** The transmittance of ITO film and ITO NWs film after annealing at 470 °C for 15 min

To verify the growth quality of the ITO NWs, transmission electron microscope (TEM) and X-ray diffraction (XRD) were measured. The optical transmittance is also measured to verify the optical property. The results are shown in Fig. 4, and shown together is the result from the ITO bulk materials.

Figure 4a is the TEM image of the NWs. Figure 4b shows the high-resolution TEM (HRTEM) recorded on the marked circle regions of Fig. 4a. The HRTEM clearly shows the high degree of crystallinity with clear lattice fringes. Spacing between the lattice fringes was found to be 0.25 nm, which is well coincided with the “d” spacing of the (400) plane of the cubic phase of In_2_O_3_. Figure 4c shows the XRD line profiles for the ITO film and NWs film. The diffraction lines show the growth structure of samples. The intensity of the two major (222) and (400) peaks at 2*θ* = 30.5° and 35.4° in ITO NWs film is more obvious than that in ITO film. The (400)/(222) ratio in ITO NWs film is more than 100 times higher than that in ITO film (inset figure in Fig. 4c), which indicates the predominant growth direction along the [100] direction [7,23–25]. Using high temperature to remove the PS spheres, the strong diffraction peaks of XRD spectra and the (400)/(222) ratio have little change, indicating that the crystal quality of ITO NWs is not affected.

ITO bulk film and ITO NWs film shown in Fig. 4d were both fabricated on quartz substrate under the same condition (10 min, 0.1 nm/s) and annealed for 15 min at 470 °C under the nitrogen ambient. The transmittance of ITO NWs has improved significantly than ITO bulk film after 400 nm. The transparency of ITO NWs film at 521 nm increased to 96 % while ITO bulk film shows improvement up to 84 %. The gap between NWs is much larger than the one in dense bulk film. Although the surface with NWs has lower reflectivity but larger absorption due to light trapping effect, the transmittance still has some improvement. At the same time, we measured the sheet resistance of both by using four-probe method, the ~16 Ω/□ for ITO film and ~200 Ω/□ for ITO NWs film. The sheet resistance of ITO NWs film is larger than the bulk material, because there are some air gaps in the intertwined NWs.

**Fig. 5:**
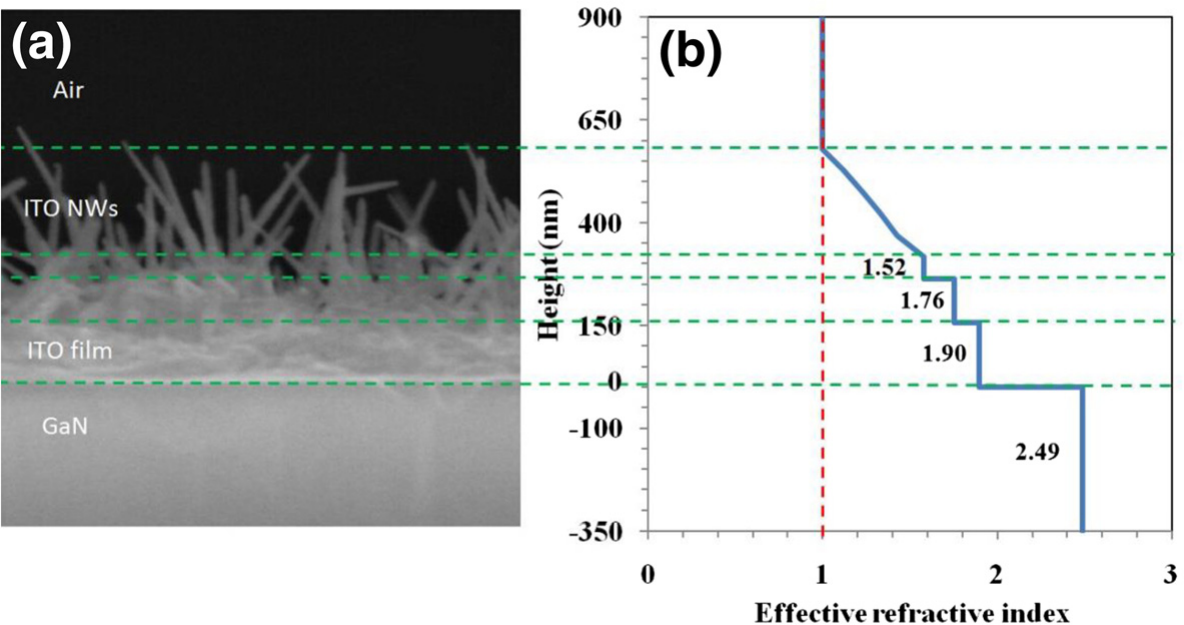
**a** The cross-sectional SEM image of the ITO NWs fabricated on GaN. **b** The effective refractive index profiles calculated based on the NWs light, which was measured from the cross-sectional SEM image

Figure 5a shows cross-sectional SEM image of the ITO NWs fabricated on GaN, showing that the NWs have a good crystal quality and a tendency to grow perpendicularly above the surface, even though all of them are not perfectly oriented on the surface. The layer is GaN, ITO film, and ITO NWs from bottom to top. In our case, the ITO NWs and air are considered to occupy an arbitrary volume, and each component has a certain volume fraction. The arbitrary volume that consists of ITO NWs and air can be regarded as an effective medium with an effective refractive index (*n*). Using ${n}_{\mathrm{eff}}=\sqrt{\left[{n}_{NW}^2{f}_{NW}+{n}_{\mathrm{air}}^2\left(1-{f}_{NW}\right)\right]}$ (*f*_NW_ is the volume fraction of ITO NWs) to calculate the effective *n* [26], the results is shown in Fig. 5b. The *n* is gradient, *n* = 1.90 with 150 nm, *n* = 1.76 with 105 nm, *n* = 1.52 with 50 nm, and the continuous gradient layer with 260 nm from *n* = 1.52 to air (*n* = 1). The effective refractive index of ITO NWs film is change from the bottom to the top with natural and gradual process, ideal material for light extraction enhancement in LEDs as transparent conducting layer.

**Fig. 6:**
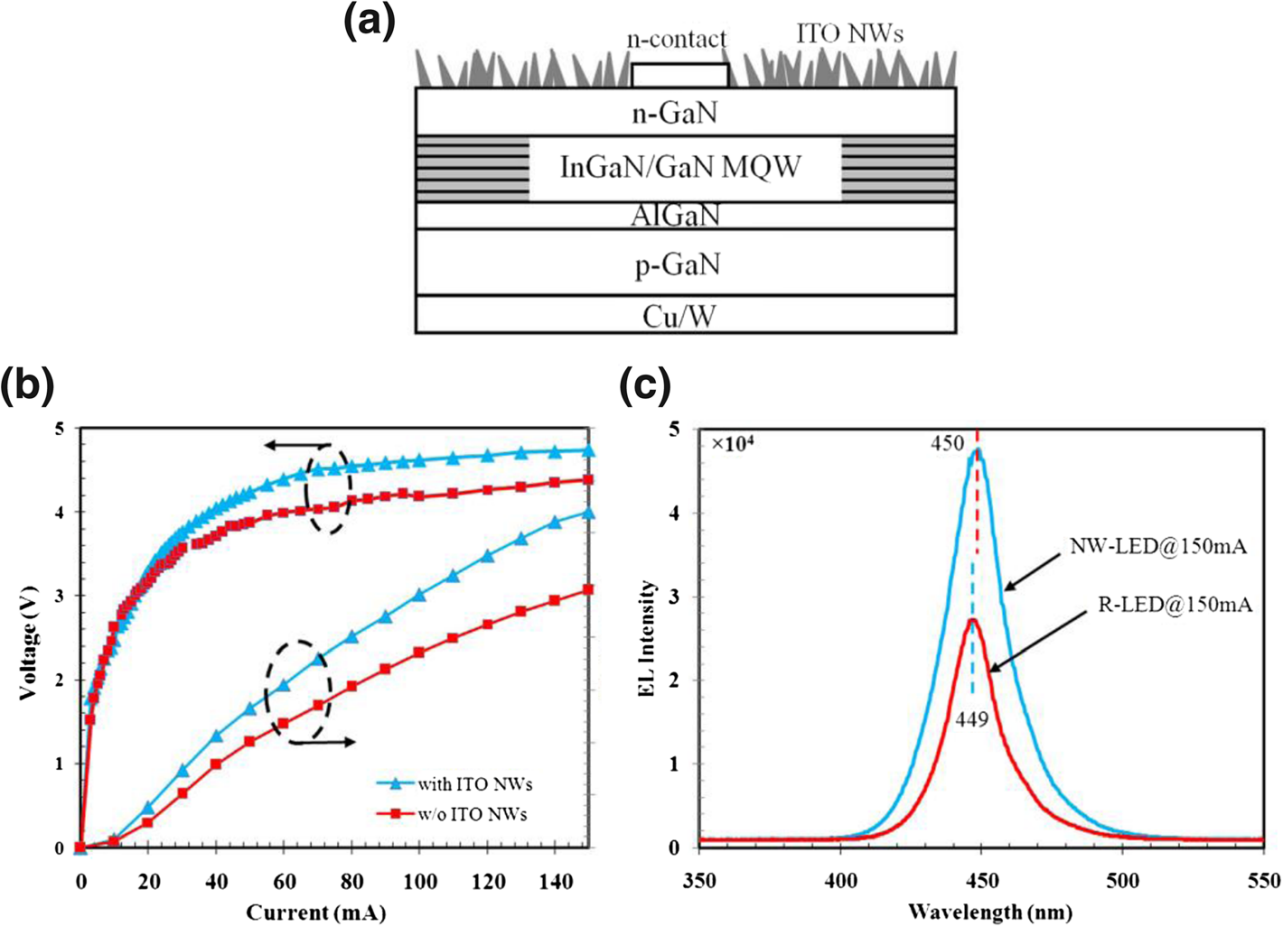
**a** The structure diagram of fabricated VLED with ITO NWs. **b** Light output power and the current-voltage (I-V) characteristics of R-VLED and NW-VLED. **c** EL spectra of R-VLED and NW-VLED at 150 mA

The ITO NWs were fabricated on the vertical LED device (VLED, *λ* = 450 nm), and the results show in Fig. 6. First, a conventional LED structure is grown on c-plane sapphire using metal-organic chemical vapor deposition (MOCVD). The epitaxial LED structure consists of five periods of InGaN/GaN QWs (5 nm well, 12 nm barrier) on a 2-μm n-type GaN:Si (n-GaN) layer. The QWs are capped by a 20-nm AlGaN:Mg electron blocking layer (EBL) followed by a 300-nm p-type GaN:Mg (p-GaN) layer. Then, p-contact Ni/Ag/Ti/Au layer is deposited onto p-GaN by electron-beam evaporation, and then, the wafer is bonding onto the Cu/W substrate. The sapphire substrate is removed using laser lift-off process [27]. After that, the n-GaN layer is exposed after etching away the undoped GaN using inductively coupled plasma (ICP). Subsequently, n-contact metal layer comprised of Al/Ti/Au is deposited on n-GaN. Prior to ITO NWs growth, standard optical lithography is used to cover the n-contact layer by photoresist, so that ITO NWs on the n-contact region could be lifted off after ITO NWs deposition. Finally, ITO NWs are fabricated through electron-beam deposition at the rate of 0.1 nm/s via PS spheres (~670 nm) for 20 min and then annealed at 470 °C for 15 min under the nitrogen condition to remove residual PS.

The electrical and optical properties of the VLED with ITO NWs (NW-VLED) are evaluated. Figure 6b shows the current-voltage (I-V) and light output power of reference VLED (R-VLED) without ITO and NW-VLED. The operation voltages of R-VLED and NW-VLED are 4.4 and 4.7 V at 150 mA, respectively. This indicates that ITO NWs do not deteriorate the electrical properties. Moreover, the relative light output power of the NW-VLED shows about 31 % enhancement at an injection current of 150 mA comparing with that of R-VLED.As discussed previously, the enhanced output power results from the improved transmittance at the surface. The random ITO NWs could be regarded as the layers with gradient refractive index due to the arbitrary volume fractions of ITO NWs and air. Thus, more photons can escape outside the VLED structure. Therefore, ITO NWs can effectively improve the light extraction efficiency.

Figure 6c shows EL spectra of R-VLED and NW-VLED at injection currents of 150 mA. There are no significant differences in the EL peak positions (at 450 nm) of the two VLEDs with the same full width at half maximum of 22 nm. However, EL intensity obtained from the NW-VLED is larger than the R-VLED at input currents of 150 mA. These results indicate that the enhancement in EL intensity of NW-VLED is owing to the increase of light extraction efficiency by ITO NWs.

## Conclusions

In summary, this work presents a method to grow ITO NWs at low temperature using PS spheres as catalyst. The use of PS spheres instead of metal catalyst has the advantages of low cost, easy removal, and low deposition temperature (280–300 °C). The fabricated ITO NWs have a better crystallinity, featuring the tendency to orientate vertically. The XRD reveals a cubic structure of the ITO NWs. The transmittance is above 90 % at a wavelength 400 nm or more, superior to the ITO bulk film. Owing to the unique morphology gradient of the ITO NWs, the effective refractive index is naturally graded from the bottom to the top, ideal for light extraction enhancement in LEDs. The ITO NWs have been used on LED devices (*λ* = 450 nm), and the light output power increased by 31 % at the current of 150 mA comparing to the one without NWs.

## Figures and Tables

**Fig. 7:**
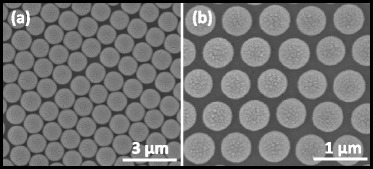
(**a**) The SEM image of PS spheres (size of 670 nm), which were coated on GaN wafer (2 in.) by self-assembly. (**b**) The PS spheres were etched by oxygen plasma for 200 s to modify the morphology and to reduce the diameter of PS spheres to ~500 nm while increasing the distance between PS spheres

## Appendix 1

The PS spheres were coated on wafer by self-assembly, and the ICP etching was used to adjust the morphology. The SEM images are shown in Appendix Fig. 7.
